# miR-1258 Attenuates Tumorigenesis Through Targeting E2F1 to Inhibit *PCNA* and *MMP2* Transcription in Glioblastoma

**DOI:** 10.3389/fonc.2021.671144

**Published:** 2021-05-17

**Authors:** Hongkun Qin, Yanping Gui, Rong Ma, Heng Zhang, Yabing Guo, Yuting Ye, Jia Li, Li Zhao, Yajing Wang

**Affiliations:** ^1^ Pathology and Patient Derived Xenograft Efficacy Evaluation Center, School of Basic Medicine and Clinical Pharmacy, China Pharmaceutical University, Nanjing, China; ^2^ Department of Anesthesiology, The First Affiliated Hospital, Nanjing Medical University, Nanjing, China; ^3^ Department of Physiology, School of Basic Medicine and Clinical Pharmacy, China Pharmaceutical University, Nanjing, China

**Keywords:** miR-1258, glioblastoma, E2F1, temozolomide, transcriptional regulation, tumorigenesis

## Abstract

MicroRNAs are a group of endogenous small non-coding RNAs commonly dysregulated in tumorigenesis, including glioblastoma (GBM), the most malignant brain tumor with rapid proliferation, diffuse invasion, and therapeutic resistance. Accumulating evidence has manifested that miR-1258 exerts an inhibitory role in many human cancers. However, the expression pattern of miR-1258 and its potential function in GBM tumorigenesis remain unclear. In this study, we reported that miR-1258 expression decreased with the ascending pathological grade of glioma, which indicated an unfavorable prognosis of patients. Functional assays revealed an inhibitory effect of miR-1258 on malignant proliferation, therapeutic resistance, migration, and invasion of GBM *in vitro*. Moreover, xenograft models also suggested a repression effect of miR-1258 on gliomagenesis. Mechanistically, miR-1258 directly targeted E2F1 in 3’-untranslated regions and attenuated E2F1-mediated downstream gene *PCNA* and *MMP2* transcriptions. Furthermore, restoration of E2F1 expression in GBM cells effectively rescued the tumor-suppressive effect of miR-1258. Our studies illustrated that miR-1258 functioned as a tumor suppressor in GBM by directly targeting E2F1, subsequently inhibiting *PCNA* and *MMP2* transcriptions, which contributed to new potential targets for GBM therapy and other E2F1-driven cancers.

## Introduction

Glioblastoma (GBM) is the most common and lethal form of glioma with high aggressiveness and low survival rate. Currently, the treatment of GBM typically consists of surgical resection, postsurgical radiotherapy, and chemotherapy. Despite those advanced therapeutic strategies, the median survival time of GBM patients is only 12–16 months, and the 5-year survival rate is less than 5% (1). Therefore, there is an urgent need to understand the molecular mechanisms and pathogenesis underlying GBM progression, which is essential to improve current therapeutic strategies for GBM.

MicroRNAs (miRNAs) are endogenous small non-coding RNAs with 20~24 nucleotides, regulating various biological processes, including cell proliferation, apoptosis, and invasion. miRNAs bind to the complementary sites in 3’-untranslated regions (3’-UTRs) of the target mRNA sequences to inhibit their translations (2). Emerging evidence has revealed that miRNAs’ aberrant expression is associated with the carcinogenesis and progression of GBM (3). In recent years, some attention has been paid to the roles of miR-1258 in human cancers. miR-1258 exerts an inhibitory role in hepatocellular carcinoma metastasis *via* targeting Smad2/3 (4). miR-1258 was reported to inhibit osteosarcoma cell proliferation by targeting AKT3 (5). Besides, miR-1258 directly targets E2F8 to regulate cell cycles and inhibit cell proliferation in colorectal cancer (6). However, its clinical relevance and molecular mechanisms in GBM are unknown.

In this study, we identified that miR-1258 expression decreased obviously in glioma patient tissue samples, comparing with normal brain tissue. Its aberrant low expression negatively correlated with the grade of glioma and indicated an unfavorable prognosis of GBM patients. Inversely, the upregulation of miR-1258 inhibited gliomagenesis *in vitro* and *in vivo*. Mechanistically, miR-1258 played a suppressive role in GBM cells by directly targeting *E2F1* mRNA 3’-UTR sequence to inhibit the E2F1-mediated downstream transcriptions of *PCNA* and *MMP2*. Our study suggested that miR-1258 might represent a promising therapeutic target in GBM.

## Materials and Methods

### Cell Lines and Primary GBM Cells Culture

Human GBM U87, U251, A172, normal human astrocytes (NHA), and 293T cell lines were obtained from the Cell Bank of the Chinese Academy of Sciences (Shanghai, China). Cell lines were authenticated using short tandem repeat profiling. The GBM cell lines and 293T cells were cultured in Dulbecco’s modified Eagle’s medium (DMEM; Gibco, USA) with 10% fetal bovine serum (FBS). NHA cells were cultured in DMEM/F-12 medium (Gibco, USA) with L-glutamine and 5% FBS. Patient-derived primary GBM cells GBM666 were freshly isolated from a surgical-resected GBM specimen. Briefly, tissues mechanically minced in prechilled DMEM/F-12 medium and digested with 2 U/mL of Dispase II (Thermofisher, USA), 0.5 mg/mL of Collagenase IV (Sigma, USA), and 10 U/mL of DNase I (Yeasen, Shanghai, China) at 37°C for 60 min. Ammonium–chloride–potassium lysing buffer was used to lyse red blood cells. After being digested, the cells were washed and passed through a 100 μm cell strainer. Finally, cells were cultured in DMEM/F-12 medium with L-glutamine, 20 ng/mL of basic fibroblast growth factor (bFGF; Thermofisher, USA), 20 ng/mL of Epidermal growth factor (EGF; Thermofisher, USA), and 15% FBS. All cells were cultured at 37°C in a humidified atmosphere with 5% CO_2_.

### Patients and Specimens

Five low-grade glioma (LGG), 28 GBM, and 5 normal brain tissue (NBT) samples between 2016 and 2020 were obtained from the Department of Neurosurgery, First Affiliated Hospital of Nanjing Medical University. Written informed consent for using the samples for this study was obtained from the patients or their family members. This research was approved by the Ethics Committee of China Pharmaceutical University.

### Public Datasets Collection

Gliomas with microarray miRNA expression data were downloaded from the Chinese Glioma Genome Atlas (CGGA) microarray database. Glioma gene expression data were downloaded from The Cancer Genome Atlas (TCGA) microarray databases. Data for miRNA-target interaction prediction were obtained from TargetScan, miRTP, miRDB, miRmap, and RNA22 databases. JASPAR and PROMO databases were used to analyze the possible promoter region and identify putative transcription factor binding sites. Data for immunohistochemical analysis were downloaded from the Human Protein Atlas database.

### Cell Transfection

miR-1258 mimic (miR-1258) and human miRNA negative control (miR-NC) were purchased from GenePharma (Shanghai, China). The E2F1 overexpression vector pEnter-E2F1 (E2F1) and pEnter empty vector (EV) were purchased from Vigenebio (Shandong, China). Cells were transfected with miRNAs or vectors using Lipofectamine 2000 (Invitrogen, USA) according to the manufacturer’s protocols.

For establishing stable miR-1258 expressing U251 cell line, the full-length coding region and miRNA flanking sequence of miR-1258 were cloned from human genomic DNA by PCR. The PCR product was transferred into the pLVX-Puro vector and packaged in 293T cells. U251 cells were transfected with the miR-NC and miR-1258 lentiviruses, and stable cell lines were selected using puromycin at 2.5 μg/mL for 7 days.

### Cell Proliferation Assays

Cell proliferation assays were performed using the CCK-8 assays (Dojindo, Japan). After transfection, cells were plated in 96-well plates at a density of 2 × 10^3^ cells per well. For drug response screening assay, transfected GBM cells were treated with different concentrations of temozolomide (TMZ, TCI, Japan) or carmustine (BCNU, Aladdin, China) for 48 h. At the indicated time points, the activity of cells was measured at OD 450 nm using SpectraMax 190 plate reader (Molecular Devices, USA).

### Colony Formation Assays

After transfection, cells were seeded in a 6-well plate at a density of 2 × 10^3^ cells per well and cultured for 7 days. The resulting colonies were washed three times with PBS and fixed with 4% formaldehyde for 15 min, finally stained with 0.5% crystal violet (Sigma, USA) for 20 min.

### Flow Cytometry Analysis

For detecting cell apoptosis, a total of 1 × 10^5^ transfected cells were seeded in a 6-well plate and treated with 500 μM TMZ for 48 h. Then pretreated cells were harvested, washed three times with prechilled PBS solution, and resuspended in a single cell suspension. The cell apoptosis analysis was performed with the Annexin V-FITC and PI Apoptosis Detection Kit (Miltenyi, Germany) according to the manufacturer’s instructions. Flow cytometric analysis was performed using MACSQuant Analyzer 10 (Miltenyi, Germany) and analyzed by Flowjo (Tree Star, USA).

### Morphological Analysis

Transfected cells were treated with 500 μM TMZ for 72 h. Then cells were viewed under an inverted microscope (Leica, Germany) at 200× magnification for morphological comparison.

### Cell Migration and Invasion Experiments

For the wound healing scratch assays, a uniform wound was made by scratching with a 200 µL pipette tip when the transfected cells reached 90% confluence in 12-well plates. Cells were maintained in a serum-free culture medium after being washed three times with PBS. After 24 h, each well was photographed under an inverted microscope (Leica, Germany) at 100× magnification. The cells protruding from the border of the scratches were counted to calculate the wound recovery rate.

For the Transwell assays, Transwell inserts (Corning, USA) were pre-coated with 20 μg/μL of Matrigel (BD Biosciences, USA), then placed in a 24-well plate. Transfected cells were resuspended at a density of 3 × 10^4^/ml in serum-free culture medium and transferred to the upper chambers. In parallel, culture medium containing 10% FBS was added to the lower chamber of each well. After incubation for 24 h, cells on the inner membrane of the upper chamber were removed with cotton swabs. Invading cells were fixed with 4% paraformaldehyde for 15 min and then stained with 0.5% crystal violet for 20 min. Three fields of invading cells in each well were captured randomly and counted under an inverted microscope (Leica, Germany) at 100× magnification.

### Protein Preparation and Western Blot

Total proteins were prepared using prechilled RIPA buffer (Thermo Fisher, USA) with proteinase inhibitor cocktail (Thermo Fisher, USA). For separating nuclear and cytoplasmic proteins, cells were harvested and lysed in nuclear and cytoplasmic extraction reagents (Keygentec, China) according to the manufacturer’s protocols. The proteins were subjected to western blot using antibodies against E2F1 (1:1000, Cell Signaling, USA), N-cadherin (1:1000, Proteintech, USA), MMP2 (1:1000, Proteintech, USA), MMP9 (1:1000, Proteintech, USA), Snail1 (1:1000, ABclonal, China), GAPDH (1:5000, Proteintech, USA), Lamin A/C (1:1000, Proteintech, USA). Relative expression levels were normalized to endogenous loading control using ImageJ software (National Institutes of Health, USA).

### RNA Extraction and Polymerase Chain Reaction (PCR)

The total RNA of cells and clinical tissues was extracted using TRIzol (Invitrogen, USA) according to the manufacturer’s instructions. One microgram of total RNA was used as a template for cDNA synthesis using a HiScript III 1st Strand cDNA Synthesis Kit (Vazyme, China). Quantitative real-time polymerase chain reaction (qRT-PCR) was performed on triplicate samples in a reaction mix of SYBR Green (Vazyme, China) with a QuantStudio 3 Real-Time PCR System (Applied Biosystems, USA). Quantification of the miR-1258 was performed with a stem-loop real-time PCR miRNA kit (Vazyme, China). The levels of mRNA were normalized to GAPDH. The levels of miR-1258 were normalized to U6 small nuclear RNA. The expressions of the indicated genes were normalized to the endogenous reference control by using the 2^−ΔΔCt^ method. Sequences of the primers used for qRT-PCR in this study are listed in **Supplementary Table S1**.

### Immunofluorescence

For immunofluorescence staining, transfected cells were fixed with 4% formaldehyde, permeabilized with 0.3% Triton X-100, and then blocked with 3% BSA for 1 hour at room temperature. After the incubation, cells were probed with E2F1 primary antibody (1:400, Cell Signaling, USA). For γ-H2A.X immunofluorescence staining, transfected cells were treated with 500 μM TMZ for 4 h, followed by fixed with 4% formaldehyde, permeabilized with 0.3% Triton X-100, and then blocked with 3% BSA for 1 hour at room temperature. After the incubation, cells were probed with Phospho-Histone H2A.X (Ser139) primary antibody (1:400, Cell Signaling, USA). After overnight incubation at 4°C, the cells were washed three times with PBS and incubated with Alexa Fluor 488-labeled Goat Anti-Rabbit IgG H&L antibodies (1:500, Abcam, USA) for 1 h at room temperature. The nuclei were stained with DAPI (Keygentec, China) and visualized with a Zeiss LSM 800 laser scanning confocal microscope (ZEISS, Germany). The γ-H2A.X foci were determined using ImageJ software (National Institutes of Health, USA).

### Dual-Luciferase Reporter Assays

Wild-type (Wt) or mutant (Mut) 3’-UTR segments of the *E2F1* gene were cloned into pmirGLO Dual-Luciferase miRNA Target Expression Vector (Promega, USA) (pmirGLO-E2F1). U251 and GBM666 cells were co-transfected with either Wt or Mut pmirGLO-E2F1 and miR-1258 or miR-NC overnight and incubated in fresh complete medium for an additional 36 h after transfection. Next, cells were harvested, and luciferase activity was measured using the Dual-Luciferase Reporter Assay Kit (Promega, USA) and normalized to Renilla luciferase activity.

Wt or Mut promoter sequences of *PCNA* and *MMP2* gene were cloned into pGL3-basic Luciferase Reporter Vector (Promega, USA) (pGL3-PCNA and pGL3-MMP2). U251 and GBM666 cells were co-transfected with either Wt or Mut pGL3-PCNA or pGL3-MMP2 plasmid, and pEnter-E2F1 or empty pEnter vector, together with pRL-TK Vector (Promega, USA) overnight, and incubated in fresh complete medium for an additional 36 h after transfection. Next, cells were harvested, and luciferase activity was measured using the Dual-Luciferase Reporter Assay Kit (Promega, USA) and normalized to Renilla luciferase activity.

### Immunohistochemistry

For immunohistochemical analysis, sections were deparaffinized and rehydrated through a descending alcohol series, followed by antigens retrieval, and endogenous peroxidase activity blocking. The sections were then incubated with primary antibodies against E2F1 (1:200, Cell Signaling, USA), PCNA (1:1000 Proteintech, USA), and MMP2 (1:400 Proteintech, USA) following by visualized with a two-step process and a DAB staining kit (ZSGB-BIO, China). Finally, slides were counterstained with hematoxylin, dehydrated, and mounted. The quantification of Immunohistochemistry staining was measured by positive-stained tumor cells. The proportion of positive-stained tumor cells was graded as follows: 1, 0%–25% positive tumor cells; 2, 25%–50% positive tumor cells; 3, 50%–75% positive tumor cells; and 4, 75% or greater positive tumor cells.

### Chromatin Immunoprecipitation (ChIP)

ChIP assays were performed with a ChIP kit (Beyotime, China) according to the manufacturer’s instructions. In brief, U251 and GBM666 cells were fixed, lysed, and sonicated. The cell lysates were clarified and precleared with Protein A/G agarose beads and salmon sperm DNA and incubated with the anti-E2F1 antibody (1:100, Cell Signaling, USA) or control rabbit IgG (1:100, Bioss, China). The immunocomplexes were sequentially washed with low-salt wash buffer, high salt wash buffer, TE buffer, and elution buffer. The eluted DNA–protein complexes were decrosslinked, purified with a DNA purification kit (Tiangen, China) according to the manufacturer’s instructions, and then subjected to PCR analysis with 3% agarose gel electrophoresis. Primer sequences are shown in **Supplementary Table S2**.

### Subcutaneous Xenograft Model

Six-week-old female athymic BALB/c nude mice were purchased from Cavans Laboratory Animals Ltd. (Changzhou, China). U251 cells stably expressing miR-1258 or miR-NC were subdivided into the miR-1258 group and miR-NC group. For establishing the subcutaneous GBM model, a total of 2 × 10^6^ U251 cells were implanted bilaterally in the axillary, respectively, per mouse. After 7 days of subcutaneous implantation, tumor volumes were measured by the formula (V= 0.5 × width^2^ × length, mm^3^) every 2 days until the tumor volume reached 1200 mm^3^. The mice were sacrificed, and subcutaneous xenografts were removed, photographed, embedded in paraffin, and sectioned for immunohistochemistry assays. All procedures were approved by the Committee on the Ethics of Animal Experiments of China Pharmaceutical University.

### Statistical Analysis

Each experiment was repeated at least three times to ensure the reliability of the results. All data were represented by mean ± standard deviation. Significant differences between the groups were estimated by Student’s t-test or one-way analysis of variance. The Kaplan-Meier curves were used to describe the survival, and the log-rank test was applied for assessing statistical significance between groups. The relationships between miR-1258 or E2F1 expression and the clinicopathological characteristics were analyzed by using the χ^2^ test. Pearson’s correlation analysis was used to assess correlations between two variables. A value of *p* < 0.05 was considered statistically significant. All statistical analyses were performed using GraphPad 8.0 (GraphPad Software, USA).

## Results

### miR-1258 Expression Decreases With Ascending Pathological Grade of Glioma and Correlates With Poor Prognosis in GBM

**Figure 1:**
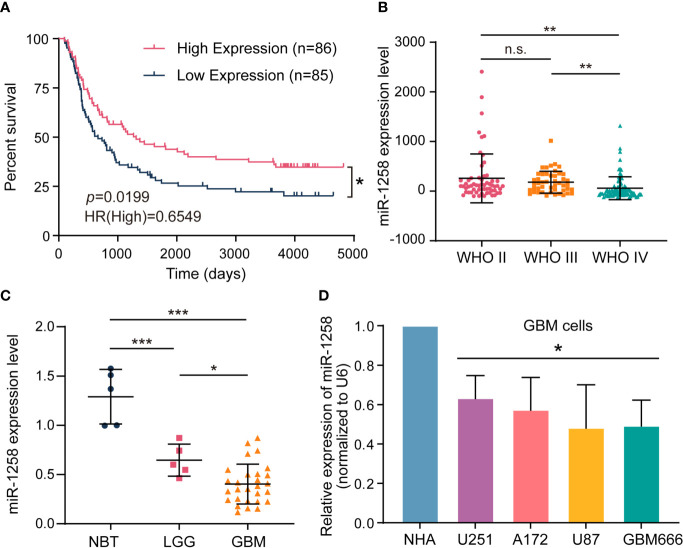
Low expressions of miR-1258 propose an unfavorable prognosis in glioma patients. **(A)** The Kaplan–Meier curve of overall survival with log‐rank test stratified by the miR-1258 level (high and low expression divided by the median expression level) in all grades of gliomas in the CGGA database; *p < 0.05 between indicated groups. **(B)** Relative miR-1258 expressions in WHO II, WHO III and WHO IV glioma patients were calculated using the CGGA database; **p < 0.01, n.s., no significance between indicated groups. **(C)** The expressions of miR-1258 in 5 NBT, 5 LGG, and 28 GBM tissues were analyzed by qRT-PCR, *p < 0.05, ***p < 0.001 between indicated groups. **(D)** miR-1258 expressions were detected in normal human astrocytes (NHA), three GBM cell lines (U251, A172, U87), and GBM666 primary GBM cells by qRT-PCR, *p < 0.05 when compared to NHA.

To investigate the role of miR-1258 in the progression of GBM, we first analyzed clinical glioma data derived from the CGGA database. Kaplan-Meier’s survival analysis revealed that patients with high miR-1258 expression had a much better overall survival rate than those with low miR-1258 expression levels in all glioma patients (*p* = 0.0199, n=171, **Figure 1A**). Moreover, the expression profiles in glioma tissues illustrated that miR-1258 expression was significantly lower in the IV grade glioma than that in the II grade and III grade gliomas (*p* < 0.01, **Figure 1B**). Besides, the analysis of correlations between miR-1258 expression level and clinicopathological characteristics revealed that low miR-1258 expression was notably associated with WHO grade (*p* < 0.001), histology (*p* < 0.001), and IDH status (*p* = 0.008) (**Supplementary Table S3**). qRT-PCR was carried out to evaluate the miR-1258 expressions in 5 LGG, 28 GBM, and 5 NBT clinical samples. miR-1258 levels in NBT were significantly higher than those in glioma specimens (*p* < 0.001). Its expression decreased with the ascending pathological grade of glioma (*p* < 0.05, **Figure 1C**). Furthermore, we found that miR-1258 expressions were downregulated in GBM cell lines and patient-derived GBM cells compared to NHAs by qRT-PCR, which is highly consistent with our above clinical sample analysis data (*p* < 0.05, **Figure 1D**). These results suggested that low expressions of miR-1258 proposed an unfavorable prognosis in glioma patients, and miR-1258 played an essential role in the progression of GBM.

### Upregulation of miR-1258 Expression Attenuates the Malignant Biological Behavior of GBM Cells

**Figure 2:**
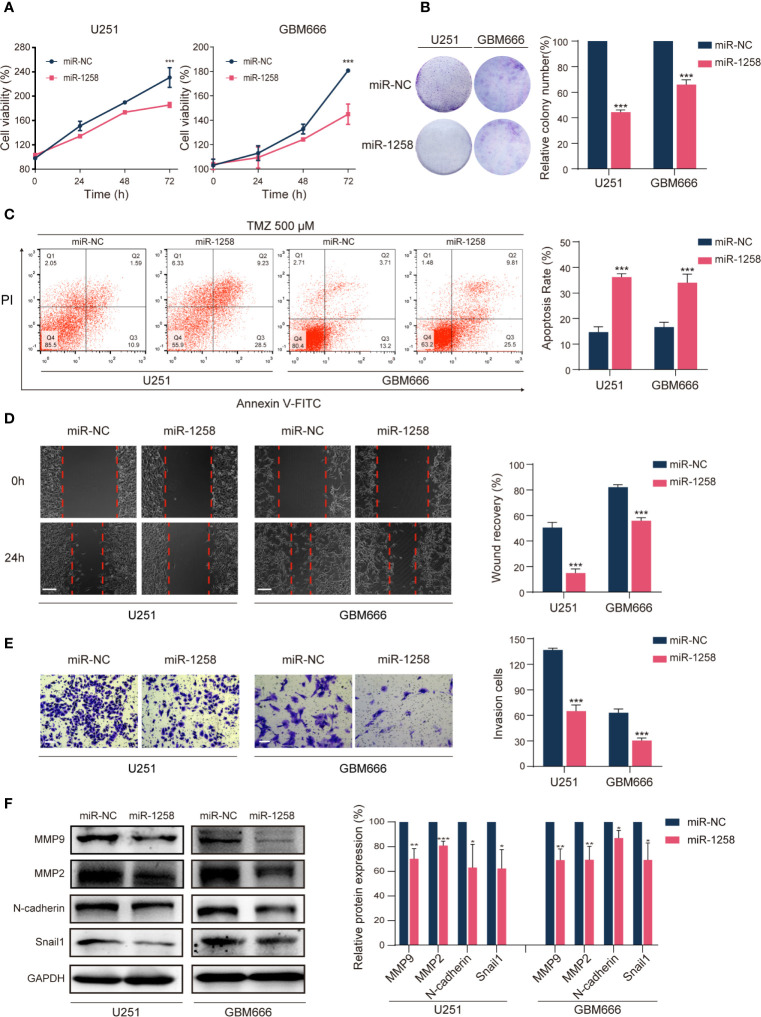
miR-1258 overexpression attenuates GBM cell tumorigenesis *in vitro*. **(A)** CCK‐8 assays detected reduced proliferation rates of U251 and GBM666 cells transfected with miR-1258 mimic after 24, 48, and 72 h; ***p < 0.001 at indicated time points, when compared to miR-NC group. **(B)** Colony formation detected reduced long-term cell viability of miR-1258 transfected GBM cells. ***p < 0.001 when compared to miR-NC group. **(C)** GBM cells were transfected with miR-1258 or miR-NC for 24 h, followed by treatment with 500 μM TMZ for 48 h, and increased apoptosis ratios were determined in miR-1258 group by flow cytometry; ***p < 0.001 when compared to miR-NC group. **(D)** Decreased migration abilities of miR-1258 transfected GBM cells were detected by wound healing scratch assays; ***p < 0.001 when compared to miR-NC group. Scale bar = 100 μm. **(E)** Matrigel invasion assays revealed the inhibitory role of miR-1258 overexpression on GBM cell invasion; ***p < 0.001 when compared to miR-NC group. Scale bar = 100 μm. **(F)** Expressions of major migration and invasion proteins were downregulated in miR-1258 transfected GBM cells detected by western blot assays; *p < 0.05, **p < 0.01, ***p < 0.001 when compared to miR-NC group. Representative images were shown and analyzed as mean ± SD from three independent experiments.

Since miR-1258 played a vital role in the progression of GBM, we next investigated the effects of miR-1258 on GBM cell proliferation, therapeutic resistance, migration, and invasion. U251 and GBM666 cells were transfected with miR-NC or miR-1258, and transfection efficiency was determined by qRT-PCR. The results confirmed that miR-1258 expressions were significantly increased in the miR-1258 group after 48 h of transfection compared to that in the miR-NC group (*p* < 0.001, **Supplementary Figure S1A**). Next, CCK-8 and colony formation assays showed that the upregulation of miR-1258 attenuated the proliferation of GBM cells (*p* < 0.001, **Figures 2A, B**). Interestingly, the upregulation of miR-1258 did not affect the apoptotic ratios of GBM cells without chemical treatment (*p* > 0.05, **Supplementary Figure S1B**). Considering that TMZ is the first-line chemotherapy regent for GBM (7), the role of miR-1258 on TMZ sensitivity of GBM cells was further investigated. Exposed to 500μM TMZ for 48 h, the flow cytometry analysis showed that miR-1258 overexpression made GBM cells more sensitive to TMZ therapy (*p* < 0.001, **Figure 2C**). The half-maximal inhibitory concentration (IC_50_) of TMZ in U251 and GBM666 cells for 48 h also indicated that miR-1258 made GBM cells become more sensitive (**Supplementary Figure S1C**). TMZ can induce DNA double-strand breaks which phosphorylate histone H2A.X at serine139 site, producing γ-H2A.X foci those are a hallmark of DNA double-strand breaks and are markedly enhanced in apoptotic cells (8). Exposed to 500μM for 4 h, the immunofluorescent analysis of γ-H2A.X foci results also illustrated that miR-1258 significantly enhanced γ-H2A.X foci formation in GBM cells induced by TMZ (*p* < 0.001, **Supplementary Figure S1D**). Finally, the results of the morphological assays showed that the cellular morphology of 500 μM TMZ-treated miR-1258 overexpressing GBM cells were damaged as compared with the miR-NC transfected GBM cells (**Supplementary Figure S1E**) at 72h. BCNU is another FDA-approved alkylating agent for the treatment of brain tumors (9). We also investigated miR-1258 on BCNU sensitivity of GBM cells, and the IC_50_ for 48 h results showed that miR-1258 overexpression also made GBM cells more sensitized to BCNU (**Supplementary Figure S1C**).

Subsequently, wound healing scratch assays identified that the migratory abilities of miR-1258 overexpressing GBM cells were significantly inhibited (*p* < 0.001, **Figure 2D**). Transwell assays also revealed that miR-1258 upregulation notably blocked the invasive abilities of GBM cells (*p* < 0.001, **Figure 2E**). Meanwhile, western blot results showed that the expressions of major migration and invasion proteins (N-cadherin, MMP2, MMP9, and Snail1) were significantly downregulated by miR-1258 in both U251 and GBM666 cells (*p* < 0.05, **Figure 2F**). In conclusion, these data indicated that miR-1258 attenuated the proliferation, migration, and invasion of GBM cells and potentiates the validity of TMZ and BCNU on GBM cells *in vitro*.

### miR-1258 Directly Targets *E2F1* mRNA in 3’-UTR

**Figure 3:**
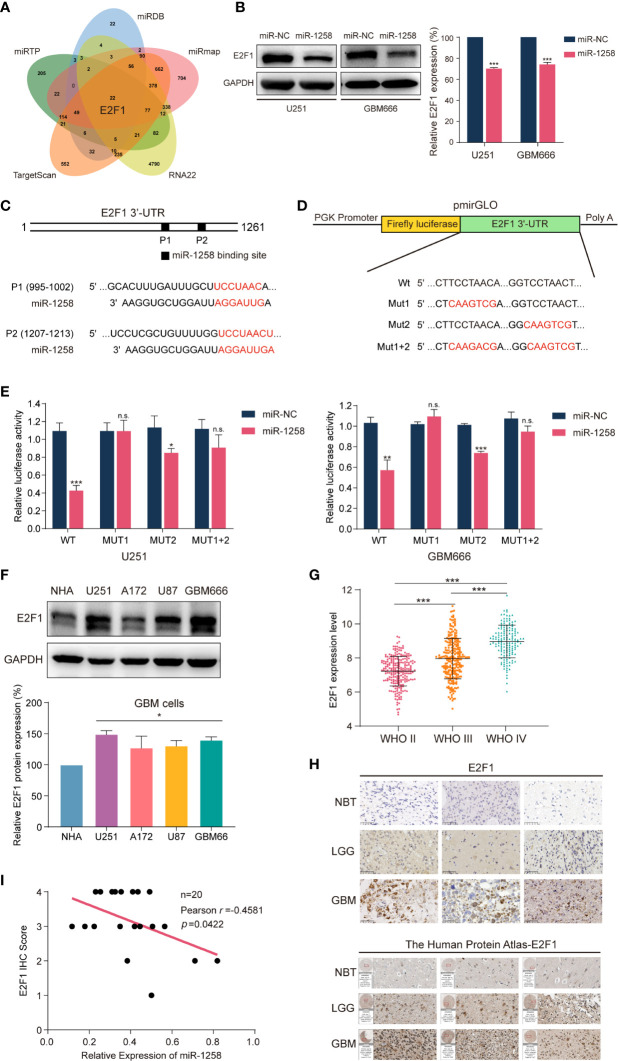
miR-1258 directly targets E2F1 in 3’-UTR and negatively regulates its expression. **(A)** The Venn diagram demonstrated that miR-1258 targeted *E2F1* mRNA intersecting five prediction databases: TargetScan, miRTP, miRDB, RNA22, and miRmap. **(B)** The decreased expressions of E2F1 protein were detected by western blot in U251 and GBM666 cells transfected with miR-1258; ***p < 0.001 when compared to miR-NC group. **(C)** Algorithms predicted two miR-1258 target sequences in the *E2F1* mRNA 3’-UTR. **(D)** The schematic diagram illustrated the construction of WT and Mut 3’-UTR sequences in pmirGLO Dual-Luciferase miRNA target expression vector. **(E)** Dual-Luciferase reporter assays were used to assess GBM cells transfected with luciferase vectors carrying WT or Mut 3’-UTR of *E2F1*, in response to miR-1258 or miR-NC; *p < 0.05, **p < 0.01, ***p < 0.001, n.s., no significance between miR-1258 and miR-NC group. **(F)** E2F1 expressions were detected by western blot in normal human astrocytes (NHA), three GBM cell lines (U251, A172, U87), and GBM666 primary GBM cells; *p < 0.05 when compared to NHA. **(G)** Relative expression data of E2F1 in 226 WHO II cases, 244 WHO III cases, and 150 WHO IV cases were analyzed using the TCGA database; ***p < 0.001 between indicated groups. **(H)** E2F1 protein expressions were determined by immunohistochemistry in NBT, LGG and GBM samples; Scale bar = 50 μm. The expressions of E2F1 were also analyzed using the Human Protein Atlas database. **(I)** The correlations between E2F1 protein expression levels and relative miR-1258 expression levels in 20 GBM samples were analyzed by Pearson’s correlation analysis; *r* = -0.4581, *p* = 0.0422.

As mentioned previously, miRNAs are endogenous non-coding RNAs composed of 20-24 nucleotides and exert their functions by binding to the 3’-UTRs of downstream target genes. To uncover the molecular mechanisms of miR‐1258 mediating anti-gliomagenesis effects, we intersected five prediction databases, including TargetScan, miRTP, miRDB, RNA22, and miRmap, and focused that *E2F1* could be one of the potential target genes of miR-1258 (**Figure 3A** and **Supplementary Table S4**).

E2F1 is a critical activator of the E2 promoter binding factor family, which controls protein expression at G1/S transition by activating related genes such as EZH2, MYC, CDK4 (10, 11). Abnormal activation of E2F1 has been studied in various tumor types in recent years. It is widely accepted that *E2F1* is an important transcription factor, which regulates cell proliferation, migration, and invasion (12–15). Meanwhile, next-generation sequencing and bioinformatics analysis have demonstrated that E2F1 is highly expressed in GBM (16). In **Figure 3B**, western blot experiments revealed that miR-1258 overexpression significantly reduced E2F1 expression in GBM cells (*p* < 0.01).

Next, Dual-Luciferase reporter assays were executed to confirm the miR-1258-binding sites in the 3’-UTR of *E2F1* mRNA. The 3’-UTR of *E2F1* exists two putative miR-1258 binding sites (position 1 995-1002, position 2 1207-1213) (**Figure 3C**). So, pmirGLO vectors containing wild-type or mutant *E2F1* 3’-UTR segments were constructed. We mutated these two binding sites individually (Mut1 and Mut2) as well as combined (Mut1+2) (**Figure 3D**). As shown in **Figure 3E**, miR-1258 significantly reduced the luciferase activity of wild-type (Wt) *E2F1* 3’-UTR in GBM cells (*p* < 0.01). The luciferase activity was also reduced in Mut2 *E2F1* 3’-UTR (*p* < 0.05), but no significant change in Mut1 and Mut1+2 (*p* > 0.05). Our results suggested that miR-1258 mainly target *E2F1* 3’-UTR in position 1 (995–1002).

Next, we measured the expression of E2F1 in different GBM cell lines by western blot. The results consistently illustrated that E2F1 expressions were higher in GBM cells than that in normal cells (*p* < 0.05, **Figure 3F**). The TCGA microarray databases illustrated that the expression levels of E2F1 increased with ascending pathological grade of glioma (*p* < 0.001, **Figure 3G**). Besides, the immunohistochemistry results of clinical GBM samples and the Human Protein Atlas database showed that E2F1 expression levels were much higher than those in NBT and LGG samples (**Figure 3H**).

We also tested the E2F1 protein expression in clinical GBM samples using western blot; same as the results of immunohistochemistry, we noticed that the expression of E2F1 was significantly higher in the glioma when compared to NBT, and increased with ascending pathological grade of glioma (*p* < 0.05, **Supplementary Figure S2A**). In addition, the analysis of correlations between E2F1 expression level and clinicopathological characteristics revealed high E2F1 expression was notably associated with age (*p* < 0.001), WHO grade (*p* < 0.001), histology (*p* < 0.001), IDH status (*p* < 0.001), 1p/19q codeletion (*p* = 0.004), *MGMT* Promoter Methylation (*p* < 0.001) and transcriptome subtype (*p* < 0.001) (**Supplementary Table S5**).

Finally, we analyzed the correlation between miR-1258 and E2F1 in the GBM samples. Pearson’s correlation analysis of our clinical GBM samples suggested that E2F1 protein expression levels were significantly negatively correlated with corresponding miR-1258 levels (n=20, *r* = -0.4581, *p* = 0.0422, **Figure 3I**). Taken together, we suggested that miR-1258 downregulated E2F1 level by directly targeting its 3’-UTR in GBM cells.

### E2F1 Is One of the Functional Targets of miR-1258 in GBM

**Figure 4:**
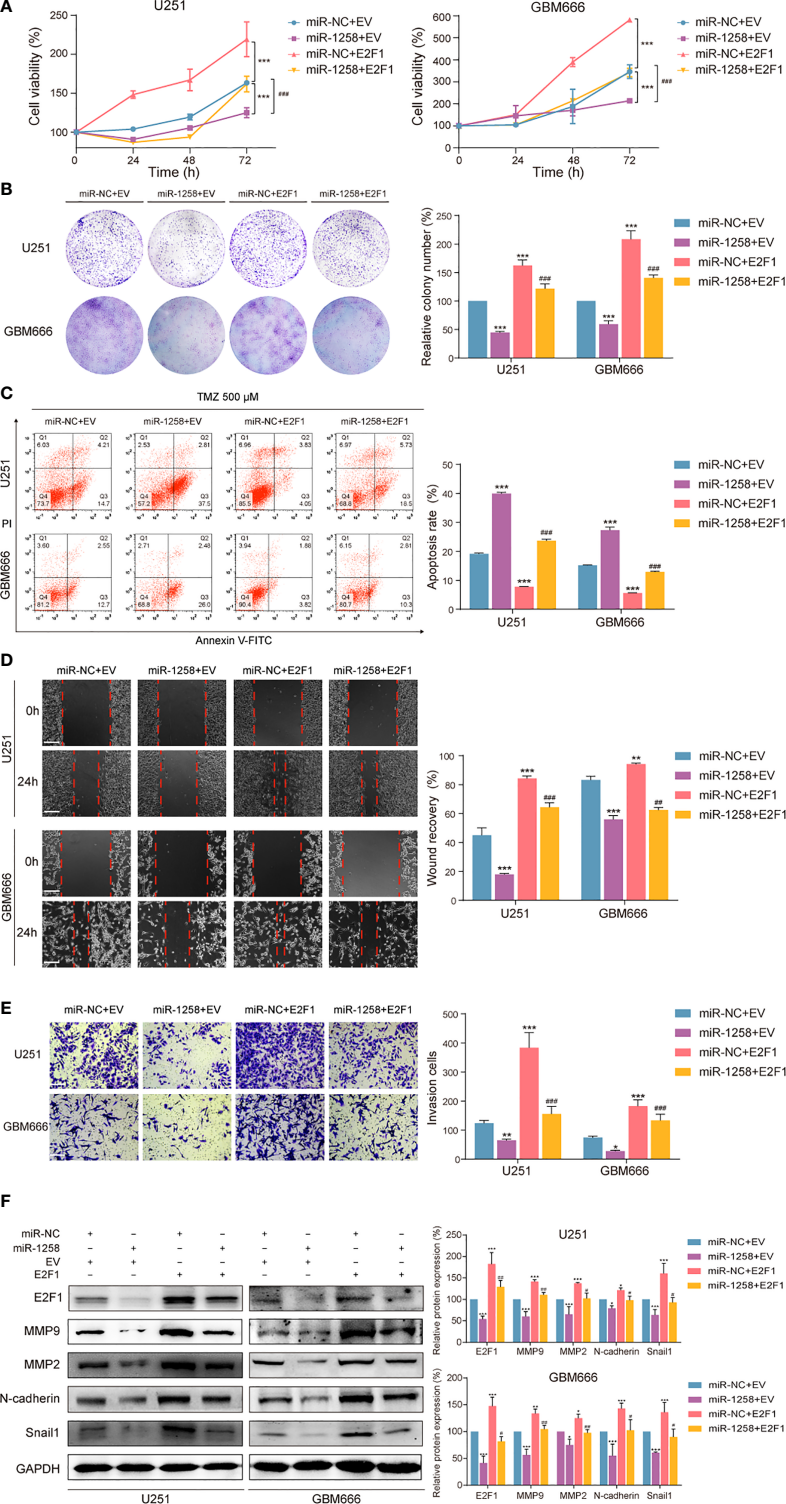
Restoration of E2F1 abrogates the inhibitory effects of miR-1258 on GBM cell proliferation, therapeutic resistance, migration, and invasion. **(A)** The viability of miR-1258 transfected U251 and GBM666 cells were rescued by E2F1 assessed by the CCK-8 assay; ***p < 0.001 versus the miR-NC+EV group. *^###^p* < 0.001, versus the miR-1258+EV group. **(B)** Colony formation assays demonstrated the long-term cell viability of miR-1258 transfected GBM cells were rescued by E2F1; ***p < 0.001 versus the miR-NC+EV group. *^###^p* < 0.001, versus the miR-1258+EV group. **(C)** After 24 h co-transfection, GBM cells were treated with 500 μM TMZ for 48 h, and then counteracted apoptosis ratios were determined in miR-1258+E2F1 group by flow cytometry; ***p < 0.001 versus the miR-NC+EV group. *^###^p* < 0.001, versus the miR-1258+EV group. **(D)** The migration abilities of miR-1258 transfected GBM cells were rescued by E2F1 assessed by wound healing scratch assays; **p < 0.01, ***p < 0.001 versus the miR-NC+EV group. *^##^p* < 0.01, *^###^p* < 0.001 versus the miR-1258+EV group. Scale bar = 100 μm. **(E)** Matrigel invasion assays revealed the rescue impact of restoration of E2F1 on GBM cells invasion; *p < 0.05, **p < 0.01 and ***p < 0.001 versus the miR-NC+EV group. *^###^p* < 0.001 versus the miR-1258+EV group. Scale bar = 100 μm. **(F)** Expressions of migration and invasion proteins were restored by E2F1 detected by western blot in GBM cells after co-transfection; *p < 0.05, **p < 0.01 and ***p < 0.001 versus the miR-NC+EV group. *^#^p* < 0.05, *^##^p* < 0.01 and *^###^p* < 0.001 versus the miR-1258+EV group.

It was concluded that E2F1 was a direct target of miR-1258, and overexpression of miR-1258 minimized various malignant biological behaviors of GBM cells above. We further verified whether E2F1 is a functional target of miR-1258. E2F1 expression vector or empty vector with either miR-1258 or miR-NC were co-transfected into U251 and GBM666 cells. Both CCK-8 and colony formation assays demonstrated that the restoration of E2F1 expression partly antagonized the anti-proliferation effects of miR-1258 (miR-1258+E2F1 vs. miR-1258+EV *p* < 0.001, **Figures 4A, B**). Flow cytometry analysis also showed that the restoration of E2F1 expression antagonized the efficacy of TMZ and reduced the pro-apoptosis effect of miR-1258 (miR-1258+E2F1 vs. miR-1258+EV *p* < 0.001, **Figure 4C**). Simultaneously, the IC_50_ of TMZ in U251 and GBM666 cells indicated that E2F1 made GBM cells become less sensitive and effectively rescued the inhibitory effect of miR-1258 (**Supplementary Figure S3A**). In the immunofluorescent analysis of γ-H2A.X foci, E2F1 enhancement modulated the TMZ-induced γ-H2A.X foci formation, which was elicited by the overexpression of miR-1258 in GBM cells (miR-1258+E2F1 vs. miR-1258+EV *p* < 0.01, **Supplementary Figure S3B**). In line with our previous results, with the distinct cellular morphology change, the restoration of E2F1 reduced the efficacy of TMZ and counteracted the inhibitory effect of miR-1258 (**Supplementary Figure S3C**).

Meanwhile, we also found that the restoration of E2F1 expression significantly promoted the migration and invasion abilities of miR-1258-expression GBM cells (miR-1258+E2F1 vs. miR-1258+EV *p* < 0.05, **Figures 4D-F**). These results showed that overexpression of E2F1 partly attenuated the anti-tumorgenesis effects of miR-1258 on malignant biological behaviors of GBM cells, indicating that E2F1 was a crucial functional target of miR-1258.

### miR-1258 Attenuates E2F1-Mediated Gliomagenesis by Downregulating Transcription of *PCNA* and *MMP2*

**Figure 5:**
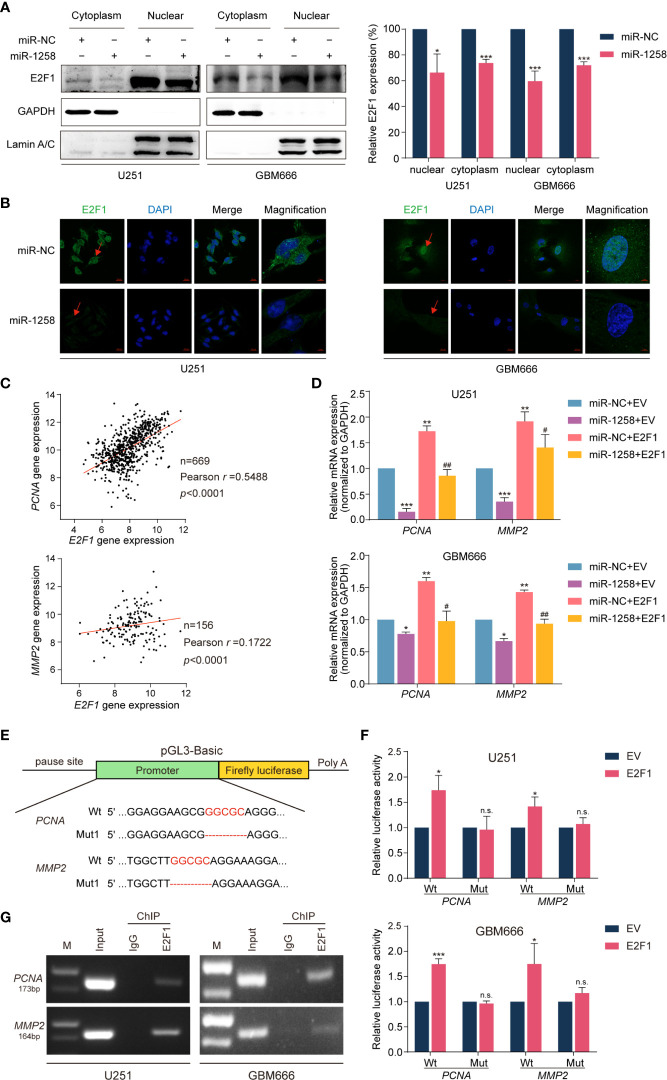
miR-1258 exerts inhibitory effects *via* transcriptional regulation of *PCNA* and *MMP2* by E2F1. **(A)** The decreased expressions of E2F1 in U251 and GBM66 cells transfected with miR-1258 in cytoplasmic and nuclear fraction were detected by western blot; *p < 0.05, ***p < 0.001 when compared to miR-NC group. **(B)** Immunofluorescence assays were applied to analyze GBM cells transfected with miR-1258 or miR-NC. Cell nuclei were stained by DAPI. Red arrows showed the nuclear expression of E2F1. Scale bar = 20 μm. In magnification, Scale bar = 5 μm. **(C)** The correlations between *E2F1* gene expression and *PCNA*, *MMP2* levels in the TCGA database were analyzed by Pearson’s correlation analysis; *r* = 0.5488, *p* < 0.0001 in *E2F1-PCNA*, *r* = 0.1722, *p* < 0.0001 in *E2F1-MMP2*. **(D)** The mRNA expressions of *PCNA* and *MMP2* in miR-1258 transfected GBM cells were restored by E2F1 using the qRT-PCR assay; *p < 0.05, **p < 0.01, ***p < 0.001 versus the miR-NC+EV group. *^#^p* < 0.05, *^##^p* < 0.01, versus the miR-1258+EV group. **(E)** The schematic diagram illustrated the construction of *PCNA* and *MMP2* WT and Mut promoter sequences in the pGL3-Basic vector. **(F)** Dual-Luciferase reporter assays were used to assess GBM cells transfected with luciferase vectors carrying WT or Mut promoter sequences of *PCNA* and *MMP2* upon transfection of an E2F1 expression vector or EV; *p < 0.05, ***p < 0.001, n.s., no significance between E2F1 and EV group. **(G)** ChIP assays were used to analyze the enrichment of E2F1 on the *PCNA* and *MMP2* promoter region in GBM cells.

All results above suggested that miR-1258 might exert inhibitory effects *via* E2F1 transcriptional regulation. Western blot results showed that miR-1258 overexpression significantly inhibited E2F1 expressions in both cytoplasm and nuclear in U251 and GBM666 cells (*p* < 0.05, **Figure 5A**). Moreover, immunofluorescence assays also demonstrated a similar tendency (**Figure 5B**).

It is widely recognized that PCNA and MMP2 widely participate in gliomagenesis (17–20). Spearman’s rank correlation analysis of the TCGA GBM database also revealed positive correlations between *E2F1* and *PCNA* or *MMP2* mRNA transcript levels (n=669, *r* =0.5488, *p* < 0.001 in *E2F1*-*PCNA*, n = 156, *r* = 0.1722, *p* < 0.001 in *E2F1*-*MMP2*, **Figure 5C**). qRT-PCR assays showed that exogenously expressed miR-1258 significantly reduced *PCNA* and *MMP2* mRNA expression in GBM cells, and the restoration of E2F1 effectively rescued the inhibitory effect of miR-1258 (miR-1258+E2F1 vs. miR-1258+EV *p* < 0.05, **Figure 5D**).

We next analyzed the promoter regions of *PCNA* and *MMP2* using JASPAR and PROMO databases and identified E2F1 binding sequences. To further support our findings, Dual-Luciferase reporter assays were executed to confirm the E2F1 binding sites in the promoter regions of *PCNA* and *MMP2* genes. pGL3-Basic vectors containing wild-type or mutant *PCNA* and *MMP2* promoter sequences were constructed (**Figure 5E**). E2F1 significantly upregulated the luciferase activities of wild-type (Wt) *PCNA* and *MMP2* promoter sequences in GBM cells (*p* < 0.05). However, there was no significant change in luciferase activity in mutation vectors (*p* > 0.05, **Figure 5F**). We confirmed that E2F1 was mainly binding to the *PCNA* promoter at position -1956~-1945 and binding to the *MMP2* promoter at position -1033~-1026, respectively. Finally, ChIP assays further showed E2F1 enriched in both *PCNA* and *MMP2* promoter regions (**Figure 5G**). These results identified that miR-1258 could partly eliminate E2F1-mediated malignant biological behaviors in GBM *via* transcriptional regulation of its downstream effectors *PCNA* and *MMP2*.

### miR-1258 Overexpression Attenuates GBM Tumorigenesis and Invasion Phenotype *In Vivo*

**Figure 6:**
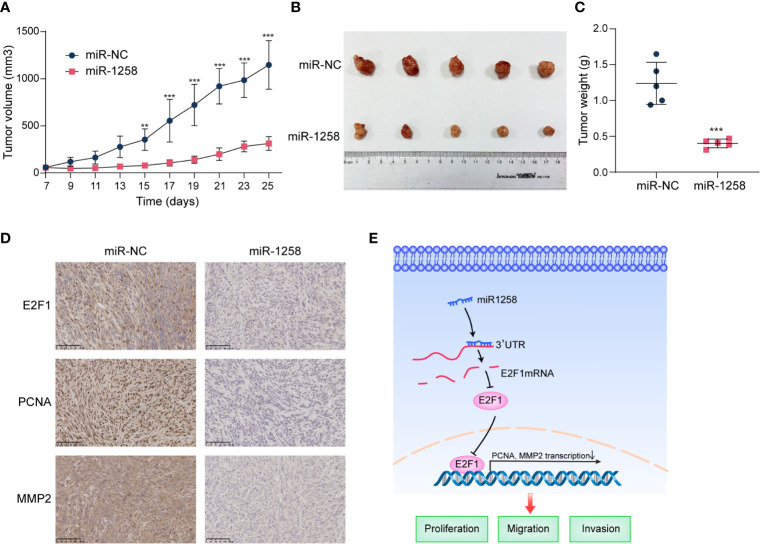
miR-1258 decreases tumorigenicity and invasion characteristics in xenograft model. **(A)** Stable‐transfected U251 cells with miR-1258 mimic or miR-NC were implanted bilaterally in the axillary, respectively, per mouse. The volumes of subcutaneous xenografts were measured from day 7 to day 25 every 2 days (V=0.5×width2×length, units: mm3, n=5); **p < 0.01, ***p < 0.001 at indicated time points, when compared to miR-NC group. **(B)** Respective images of subcutaneous xenografts were shown. **(C)** The tumor weights were decreased in miR-1258 group; ***p < 0.001 between indicated groups. **(D)** The expressions of E2F1, PCNA, and MMP2 significantly diminished in miR-1258 group detected by immunohistochemical analysis. Representative images were shown; Scale bar, 100 μm. **(E)** The mechanistic scheme illustrated that miR-1258 attenuates tumorigenesis through targeting E2F1 to inhibit *PCNA* and *MMP2* transcription in glioblastoma.

Considering the significant inhibitory effects of miR-1258 on GBM cells *in vitro*, we further investigate the anti-tumorgenesis effects of miR-1258 in a subcutaneous xenograft GBM model. Results manifested that the growth of subcutaneous tumors was significantly inhibited by miR-1258 overexpression (*p* < 0.01, **Figures 6A–C**). Moreover, immunohistochemistry suggested that the miR-1258 overexpressing group had lower expressions of E2F1, PCNA, and MMP2 (**Figure 6D**), consistent with our results *in vitro*. The animal experiment results confirmed that miR-1258 exerted an inhibitory effect on gliomagenesis by repressing E2F1 expression.

## Discussion

The existing therapeutic approach to GBM consists of surgical resection followed by concurrent radiotherapy and chemotherapy. Though those approach has improved survival, almost GBM patients still succumb. Effective treatment options for GBM are not well established (21). Elucidating the molecular basis of GBM progression could contribute to novel therapies to block GBM progression.

Accumulating evidence has indicated that abnormal expression of miRNAs is closely associated with the tumorigenesis of human cancers (22–24), which can serve as molecular biomarkers for diagnosis, prognosis, and treatment for GBM (25). Similarly, our results first reported that miR‐1258 expression decreased with the ascending pathological grade of glioma in both the CGGA database and clinical samples. Notably, patients with high miR-1258 expression had much better overall survival, which suggested miR-1258 function as a potential diagnostic and prognostic biomarker for glioma patients, especially the GBM patients. In previous studies, miR-1258 has been demonstrated an inhibitory role in many cancer types (4, 5, 26). Here, we also noticed that miR-1258 acted as a tumor‐suppressive miRNA on GBM proliferation, therapeutic resistance, migration, and invasion *in vitro* and *in vivo* by functional experiments. The expression of miRNAs in serum is stable (27), and peripheral blood is easy to collect from patients. miR-1258 may therefore be a potential tumor marker and help the development of valuable tools for early diagnosis of gliomas in clinical practice for neuro-oncologists and pathologists. Interestingly, we found that miR-1258 affected the apoptotic rate of GBM cells when TMZ existed. As listed in **Supplementary Table S4**, we also found that *PARP3*, *ABCG1* and *BCL2* were potential targets of miR-1258, whose expression could be downregulated by miR-1258. Recently studies have revealed that combining PARP3 inhibitors with TMZ can potentiate the validity of TMZ in SW620 cell subcutaneous xenograft models (28). ABCG1 has been demonstrated highly expressed in GBM (29, 30). As a member of the ATP-binding cassette transporters, ABCG1 induces drug resistance in many cancers, including hepatocellular carcinoma and osteosarcoma (31, 32), so we speculated that ABCG1 may also participate in TMZ resistance in GBM. BCL-2, one of the most common anti-apoptotic proteins, encoded by the *BCL2* gene, has been widely reported to involve in TMZ resistance (33, 34). Hence, further study on the participation and the effect of miR-1258 on TMZ sensitivity is warranted.

E2F1, as a member of the E2F family, is an essential transcription factor, which involves in the regulation of multiple biological processes of cancers, including cell cycle, programmed cell death, DNA damage, and self-renew of cells (35, 36). Activation of E2F1 can also initiate the target genes transcription to maintain the progression of the cell cycles and induce the progression of anti-apoptosis and cell invasion in cancers (15, 37). Abnormal overexpression of E2F1 has also been reported in glioma (16, 38). Interestingly, there has been increasing attention that miRNAs post-transcriptionally regulate E2F1 expression, including miR-205-5p, miR-342-3p, miR-93, and miR-598 (39–41). To better comprehend the tumor‐suppressive function of miR-1258 mechanistically, we identified E2F1 as one of the direct and functional targets for miR-1258 in GBM. Dual-Luciferase reporter assays demonstrated miR-1258 directly binding to *E2F1* 3’-UTR, thereby inhibiting E2F1 expression, consistent with our results that E2F1 expressed highly in GBM tissues as well as GBM cell lines. By contrast, the restoration of E2F1 reversed GBM cell proliferation inhibition, therapeutic resistance, migration, and invasion mediated by enhanced expression of miR-1258. Our study results indicated that the miR‐1258/E2F1 axis represented a critical regulation mechanism underlying the molecular pathogenesis of GBM. Very recently, Peng X et al. has reported that miR-1258 overexpression inhibits cell proliferation, invasion and migration by targeting the E2F1 and altering AKT and P53 signal pathway in cervical cancer (42), which also highlights a wide regulatory network of miR‐1258/E2F1 axis in cancers.

*PCNA*, which encodes proliferating cell nuclear antigen, is an essential factor in DNA replication and many other cancer cell processes, especially in cell proliferation (43). Matrix metalloproteinase 2, an enzyme encoded by the *MMP2* gene, is involved in degrading type IV collagen. It is widely recognized that matrix metalloproteinase-2 mediated basement membrane degradation is positively linked with the migration, invasion, and metastasis of cancers (44). Only a few studies have reported that E2F1 stimulates transcription of the *PCNA* and *MMP2* (45, 46). However, the transcriptional regulations of E2F1 on *PCNA* and *MMP2* genes in GBM remain unclear. In this study, we demonstrated that E2F1 directly bound to the promoter regions of *PCNA* and *MMP2* to regulate their transcriptional activity, facilitating the expression of these gliomagenesis genes, finally attenuating the inhibitory effects of miR-1258 on cell proliferation, therapeutic resistance, migration, and invasion in GBM cells. These findings also indicated that E2F1 exerted a crucial role in the malignant behavior of GBM and could serve as a potential diagnostic marker and therapeutic target in GBM. However, it is still unclear whether E2F1 participates in the regulation of other downstream effectors in gliomagenesis. Hence, this issue deserves to be clarified.

Our findings collectively highlighted the novel evidence of a crucial link between miR-1258 and the tumorigenesis of human GBM. miR-1258 played an inhibitory role in the progression of GBM and functioned as a tumor suppressor by down-regulating E2F1 expression and attenuated E2F1-mediated downstream gene *PCNA* and *MMP2* transcriptions (**Figure 6E**). To date, miRNA-based therapies are still in the initial stages (25). Our novel findings establish the role of miR-1258 as a potential diagnostic marker and a therapeutic target in GBM.

## Data Availability Statement

The original contributions presented in the study are included in the article/**Supplementary Material**. Further inquiries can be directed to the corresponding authors.

## Ethics Statement

The studies involving human participants were reviewed and approved by Ethics Committee of China Pharmaceutical University. The patients/participants provided their written informed consent to participate in this study. The animal study was reviewed and approved by Ethics Committee of China Pharmaceutical University.

## Author Contributions

LZ and YW conceived and supervised the research. HQ and YanG designed, performed all the experiments, and interpreted data. RM provided clinical samples. HZ and YaG helped conduct animal experiments. YY and JL helped conduct immunohistochemistry experiments. HQ wrote the manuscript. HQ, LZ, and YW revised the manuscript. All authors contributed to the article and approved the submitted version.

## Funding

This study was supported by the National Natural Science Foundation of China (No. 81773774 and No. 81872903), the “Double First-Class” University project of China Pharmaceutical University (CPU2018GY03 and CPU2018GY37), and the Postgraduate Research & Practice Innovation Program of Jiangsu Province (KYCX20-0656).

## Conflict of Interest

The authors declare that the research was conducted in the absence of any commercial or financial relationships that could be construed as a potential conflict of interest.

## Abbreviations

miRNA, microRNA; GBM, glioblastoma; 3’-UTR, 3’-untranslated regions; NHA, normal human astrocytes; DMEM, Dulbecco’s modified Eagle’s medium; FBS, fetal bovine serum; LGG, low-grade glioma; NBT, normal brain tissue; CGGA, The Chinese Glioma Genome Atlas; TCGA, The Cancer Genome Atlas; miR-1258, miR-1258 mimic; NC, negative control; EV, empty vector; PCR, polymerase chain reaction; qRT-PCR, quantitative real-time polymerase chain reaction; Wt, wild-type; Mut, mutant; ChIP, chromatin immunoprecipitation; TMZ, temozolomide; BCNU, carmustine.
