# Supplementary material for: miR-1258 Attenuates Tumorigenesis Through Targeting E2F1 to Inhibit PCNA and MMP2 Transcription in Glioblastoma
Source: Front Oncol. 2021 May 17;11:671144. doi: 10.3389/fonc.2021.671144 (PMC8166228; doi:10.3389/fonc.2021.671144)
Supplement: Supplementary file 1 [file Image_1.pdf]

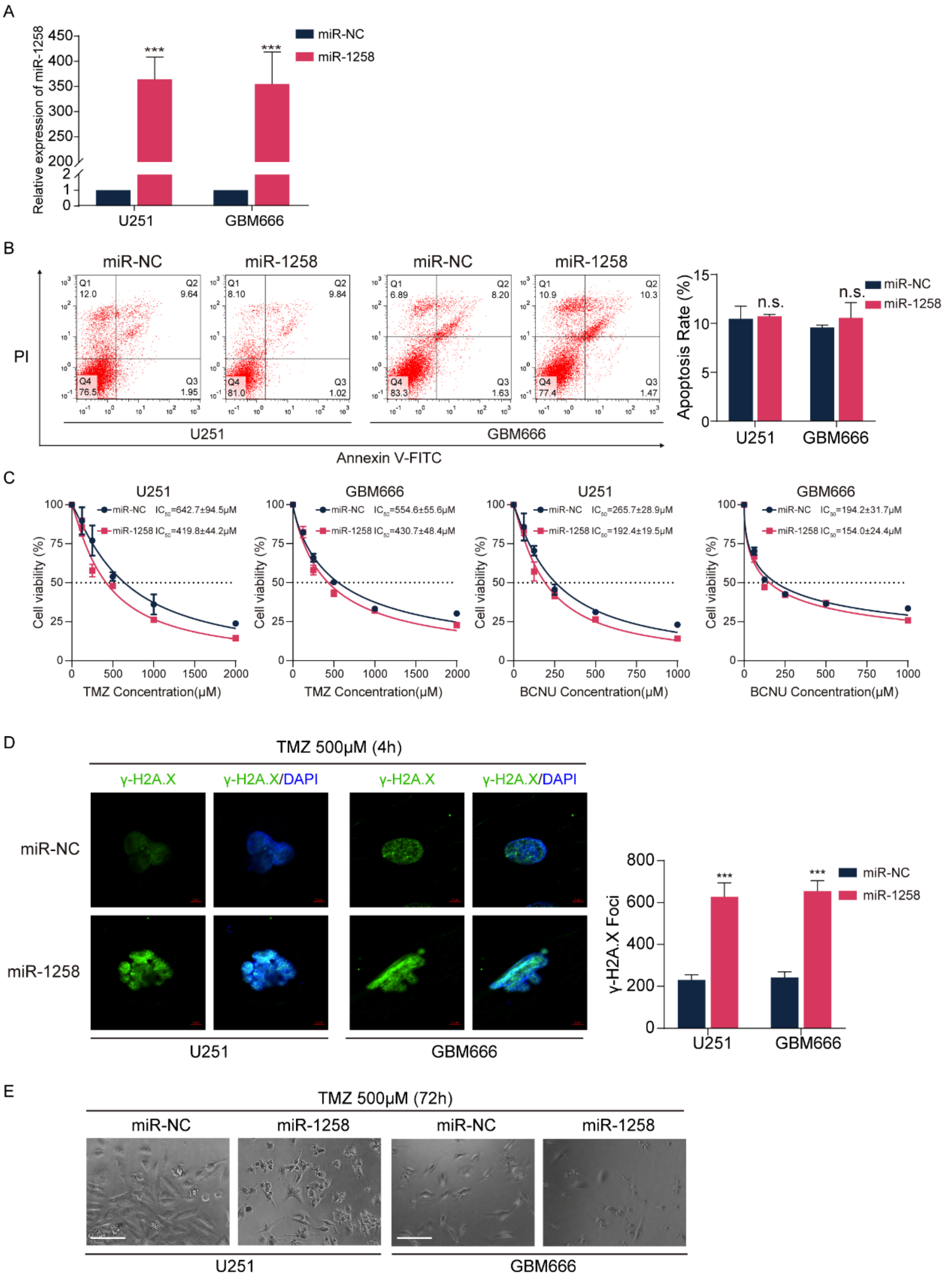

Supplementary Figure S1. (A) The expressions of miR-1258 in U251 and GBM666 cells transfected with miR-1258 mimic or miR-NC were quantified by qRT-PCR; $***p < 0.001$ when compared to miR-NC group. **(B)** GBM cells were transfected with miR-1258 mimic or miR-NC for 24 h, and no significant apoptosis ratio changes were determined by flow cytometry; n.s., no significance when compared to miR-NC group. **(C)** After transfection for 24 h, GBM cells were treated with TMZ at concentrations of

0, 125, 250, 500, 1000, 2000 μM or BNCU at concentrations of 0, 62.5, 125, 250, 500, 1000 μM for 48h, respectively. The IC_{50} value were assessed by the CCK8 assay. **(D)** GBM cells were transfected with miR-1258 or miR-NC for 24 h, followed by treatment with 500 μM TMZ for 4h, and then more γ-H2A.X foci were detected in miR-1258 group by laser scanning confocal microscope. Scale bar = 5 μm; $^{***}p < 0.001$ when compared to miR-NC group. **(E)** GBM cells were transfected with miR-1258 or miR-NC for 24 h, followed by treatment with 500 μM TMZ for 72h, and cellular morphology of TMZ-treated miR-1258 over-expressing GBM cells was damaged. Scale bar = 150 μm. Representative images were shown and analyzed as mean ± SD from three independent experiments.
