# Supplementary material for: miR-1258 Attenuates Tumorigenesis Through Targeting E2F1 to Inhibit PCNA and MMP2 Transcription in Glioblastoma
Source: Front Oncol. 2021 May 17;11:671144. doi: 10.3389/fonc.2021.671144 (PMC8166228; doi:10.3389/fonc.2021.671144)
Supplement: Supplementary file 2 [file Image_2.pdf]

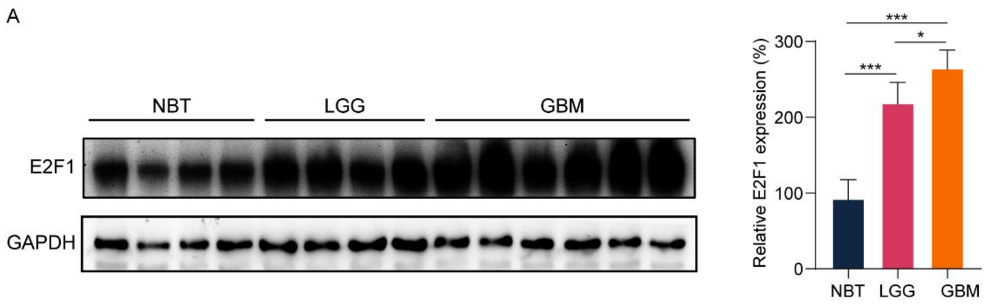

Supplementary Figure S2. (A) The expressions of E2F1 pretein were detected by western blot in 4 NBT, 4 LGG and 6 GBM tissues; *p < 0.05, ***p < 0.001 between indicated groups.
