# Supplementary material for: miR-1258 Attenuates Tumorigenesis Through Targeting E2F1 to Inhibit PCNA and MMP2 Transcription in Glioblastoma
Source: Front Oncol. 2021 May 17;11:671144. doi: 10.3389/fonc.2021.671144 (PMC8166228; doi:10.3389/fonc.2021.671144)
Supplement: Supplementary file 3 [file Image_3.pdf]

A

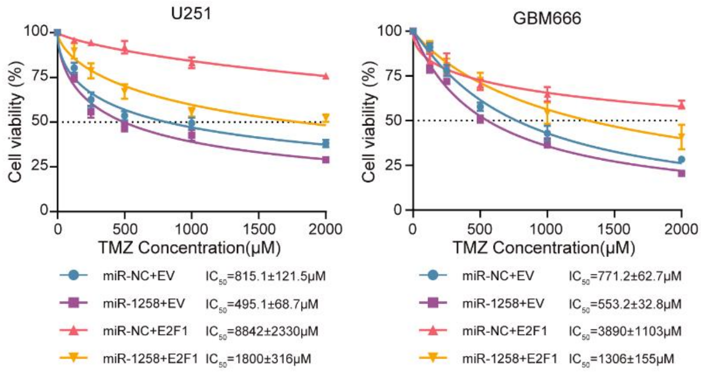
U251
GBM666
Cell viability (%)
TMZ Concentration(μM)
miR-NC+EV IC50=815.1±121.5μM
miR-1258+EV IC50=495.1±68.7μM
miR-NC+E2F1 IC50=8842±2330μM
miR-1258+E2F1 IC50=1800±316μM
miR-NC+EV IC50=771.2±62.7μM
miR-1258+EV IC50=553.2±32.8μM
miR-NC+E2F1 IC50=3890±1103μM
miR-1258+E2F1 IC50=1306±155μM

B

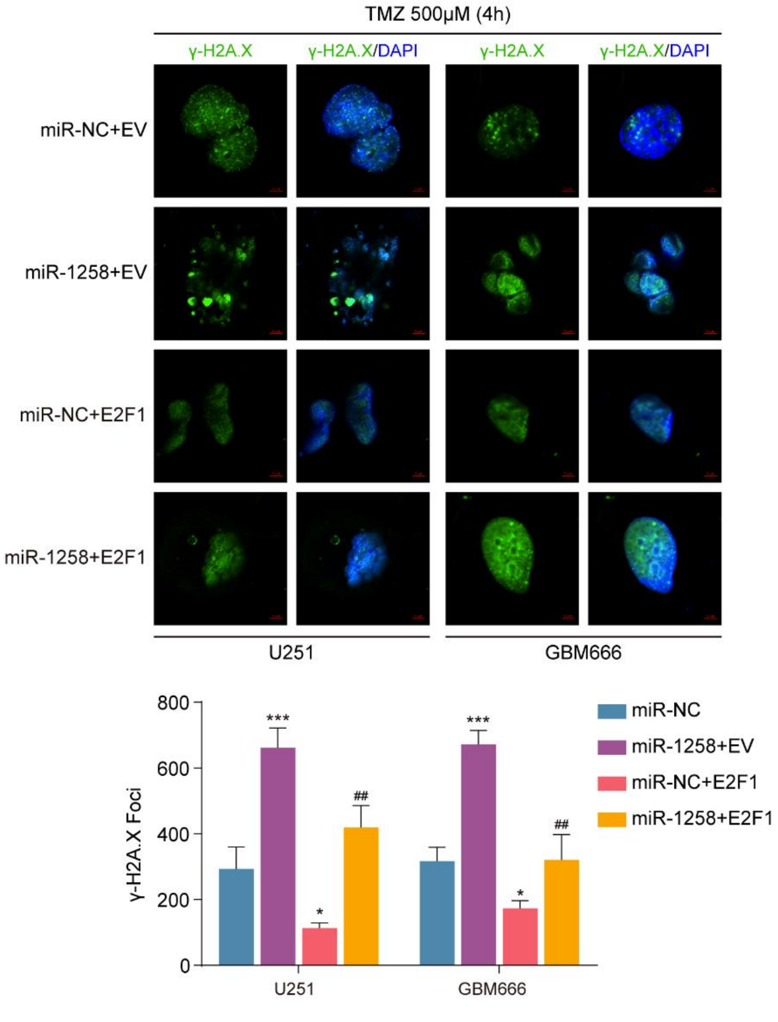
TMZ 500μM (4h)
γ-H2A.X
γ-H2A.X/DAPI
miR-NC+EV
miR-1258+EV
miR-NC+E2F1
miR-1258+E2F1
U251
GBM666
γ-H2A.X Foci
miR-NC
miR-1258+EV
miR-NC+E2F1
miR-1258+E2F1

C

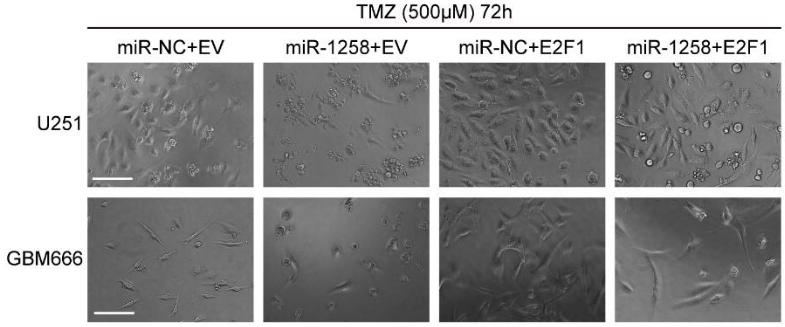
TMZ (500μM) 72h
miR-NC+EV
miR-1258+EV
miR-NC+E2F1
miR-1258+E2F1
U251
GBM666

**Supplementary Figure S3. (A)** U251 and GBM66 cells were transfected with E2F1 expression vector or EV in the presence of miR-1258 mimic or miR-NC for 24h, followed by treatment with TMZ at concentrations of 0, 125, 250, 500, 1000 and 2000 μM for 48h, respectively. The $IC_{50}$ value were assessed by the CCK8 assay. **(B)** After 24h co-transfected, GBM cells were treated with 500 μM TMZ for 4h, and the number of γ-H2A.X foci were decreased by E2F1; $^{*}p < 0.05$ and $^{***}p < 0.001$ versus the miR-NC+EV group. $^{\#\#}p < 0.01$ versus the miR-1258+EV group. Scale bar=5μm. **(C)** After 24h co-transfected, GBM cells were treated with 500 μM TMZ for 72h, and the damage of cellular morphology in TMZ-treated miR-1258 overexpressing GBM cells were rescued by E2F1. Scale bar = 150 μm. Representative images were shown and analyzed as mean ± SD from three independent experiments.
