# Supplementary material for: miR-1258 Attenuates Tumorigenesis Through Targeting E2F1 to Inhibit PCNA and MMP2 Transcription in Glioblastoma
Source: Front Oncol. 2021 May 17;11:671144. doi: 10.3389/fonc.2021.671144 (PMC8166228; doi:10.3389/fonc.2021.671144)
Supplement: Supplementary file 4 [file Table_1.docx]

Supplementary Table S1. The sequences of primers used in qRT-PCR.

| Primers | Sequences (5’–3’) |
| --- | --- |
| miR-1258-F | CGCGCGAGTTAGGATTAGGTC |
| U6-F | CTCGCTTCGGCAGCACA |
| Universal-R | GTCGTATCCAGTGCAGGGTCCGAGGTATTCGCACTGGATACGAC |
| PCNA-F | CAAGTAATGTCGATAAAGAGGAGG |
| PCNA-R | GTGTCACCGTTGAAGAGAGTGG |
| MMP2-F | AGCGAGTGGATGCCGCCTTTAA |
| MMP2-R | CATTCCAGGCATCTGCGATGAG |
| GAPDH-F | GTCTCCTCTGACTTCAACAGCG |
| GAPDH-R | ACCACCCTGTTGCTGTAGCCAA |
