# Supplementary material for: miR-1258 Attenuates Tumorigenesis Through Targeting E2F1 to Inhibit PCNA and MMP2 Transcription in Glioblastoma
Source: Front Oncol. 2021 May 17;11:671144. doi: 10.3389/fonc.2021.671144 (PMC8166228; doi:10.3389/fonc.2021.671144)
Supplement: Supplementary file 5 [file Table_2.docx]

Supplementary Table S2. The sequences of primers used in ChIP.

| Primers | Sequences (5’–3’) |
| --- | --- |
| PCNA-F | GCGACGTCACCACGCTGTC |
| PCNA-R  MMP2-F  MMP2-R | TGCGGCCGGGTTCAGGAG  CCATCACAGCTTATCTCTCAACTG  CACGAATTCCCCACTCACTC |
