# Supplementary material for: miR-1258 Attenuates Tumorigenesis Through Targeting E2F1 to Inhibit PCNA and MMP2 Transcription in Glioblastoma
Source: Front Oncol. 2021 May 17;11:671144. doi: 10.3389/fonc.2021.671144 (PMC8166228; doi:10.3389/fonc.2021.671144)
Supplement: Supplementary file 6 [file Table_3.docx]

Supplementary Table S3. Correlation between miR-1258 expression in 198 glioma patients and clinicopathological characteristics from CGGA database

| Characteristic | Number | No. Low expression | No. High expression | *p* |
| --- | --- | --- | --- | --- |
| **Gender** |  |  |  | 0.558 |
| Female | 75 | 35 | 40 |  |
| Male | 123 | 64 | 59 |  |
| **Age** |  |  |  | 0.569 |
| <40 | 93 | 44 | 49 |  |
| ≥40 | 104 | 54 | 50 |  |
| **WHO grade** |  |  |  | < 0.001* |
| Ⅱ | 60 | 21 | 39 |  |
| Ⅲ | 47 | 15 | 32 |  |
| Ⅳ | 91 | 63 | 28 |  |
| **Histology** |  |  |  | < 0.001* |
| A | 46 | 15 | 31 |  |
| AA | 28 | 9 | 19 |  |
| AO | 14 | 5 | 9 |  |
| AOA | 5 | 1 | 4 |  |
| O | 13 | 5 | 8 |  |
| GBM | 91 | 63 | 28 |  |
| **IDH status** |  |  |  | 0.008* |
| Wildtype | 106 | 62 | 44 |  |
| Mutant | 81 | 31 | 50 |  |
| **1p/19q codeletion** |  |  |  | >0.999 |
| Codel | 7 | 2 | 5 |  |
| Non-codel | 19 | 7 | 12 |  |
| ***MGMT* Promoter Methylation** |  |  |  | 0.643 |
| methylation | 63 | 31 | 32 |  |
| un-methylated | 122 | 65 | 57 |  |

A astrocytoma, AA anaplastic astrocytoma, AO anaplastic oligodendrocytoma, AOA anaplastic oligodendrocytoma, O oligodendrocytoma, GBM glioblastoma.
