# Supplementary material for: miR-1258 Attenuates Tumorigenesis Through Targeting E2F1 to Inhibit PCNA and MMP2 Transcription in Glioblastoma
Source: Front Oncol. 2021 May 17;11:671144. doi: 10.3389/fonc.2021.671144 (PMC8166228; doi:10.3389/fonc.2021.671144)
Supplement: Supplementary file 8 [file Table_5.docx]

Supplementary Table S5. Correlation between E2F1 expression in 609 glioma patients and clinicopathological characteristics from TCGA database

| Characteristic | Number | No. Low expression | No. High expression | *p* |
| --- | --- | --- | --- | --- |
| **Gender** |  |  |  | 0.681 |
| Female | 254 | 130 | 124 |  |
| Male | 355 | 175 | 180 |  |
| **Age** |  |  |  | < 0.001* |
| <50 | 339 | 224 | 115 |  |
| ≥50 | 270 | 80 | 190 |  |
| **WHO grade** |  |  |  | < 0.001* |
| Ⅱ | 226 | 174 | 52 |  |
| Ⅲ | 244 | 112 | 132 |  |
| Ⅳ | 150 | 22 | 128 |  |
| **Histology** |  |  |  | < 0.001* |
| oligodendrocytoma | 191 | 113 | 78 |  |
| oligoastrocytomas | 130 | 85 | 45 |  |
| astrocytoma | 194 | 114 | 80 |  |
| glioblastoma | 152 | 22 | 130 |  |
| **IDH status** |  |  |  | < 0.001* |
| Wildtype | 233 | 62 | 171 |  |
| Mutant | 429 | 331 | 98 |  |
| **1p/19q codeletion** |  |  |  | 0.004* |
| Codel | 169 | 101 | 68 |  |
| Non-codel | 494 | 232 | 262 |  |
| ***MGMT* Promoter Methylation** |  |  |  | < 0.001* |
| methylation | 477 | 264 | 213 |  |
| un-methylated | 161 | 55 | 106 |  |
| **Transcriptome subtype** |  |  |  | < 0.001* |
| proneural | 238 | 142 | 96 |  |
| neural | 111 | 78 | 33 |  |
| mesenchymal | 96 | 35 | 61 |  |
| classical | 86 | 10 | 76 |  |
